# Waste level detection and HMM based collection scheduling of multiple bins

**DOI:** 10.1371/journal.pone.0202092

**Published:** 2018-08-29

**Authors:** Fayeem Aziz, Hamzah Arof, Norrima Mokhtar, Noraisyah M. Shah, Anis S. M. Khairuddin, Effariza Hanafi, Mohamad Sofian Abu Talip

**Affiliations:** Faculty of Engineering, University of Malaya, Kuala Lumpur, Malaysia; Nanjing University of Information Science and Technology, CHINA

## Abstract

In this paper, an image-based waste collection scheduling involving a node with three waste bins is considered. First, the system locates the three bins and determines the waste level of each bin using four Laws Masks and a set of Support Vector Machine (SVM) classifiers. Next, a Hidden Markov Model (HMM) is used to decide on the number of days remaining before waste is collected from the node. This decision is based on the HMM’s previous state and current observations. The HMM waste collection scheduling seeks to maximize the number of days between collection visits while preventing waste contamination due to late collection. The proposed system was trained using 100 training images and then tested on 100 test images. Each test image contains three bins that might be shifted, rotated, occluded or toppled over. The upright bins could be empty, partially full or full of garbage of various shapes and sizes. The method achieves bin detection, waste level classification and collection day scheduling rates of 100%, 99.8% and 100% respectively.

## Introduction

An effective Solid Waste Management (SWM) system is crucial to manage increasing solid waste generated by a growing population and maintain cleanliness of the waste sites [1]. However, a proper SWM is expensive as it requires funding, personnel, equipment, infrastructure and an efficient operation strategy. To sustain the operation of a modern SWM, the support and involvement of the authority and the public are crucial [2]. An integrated waste management system for an urban population requires careful planning of the movement of waste materials from the generation points to the treatment and disposal sites [3]. Various deterministic waste management models have been proposed but most of them are dedicated to either the logistics of trucks or land fill strategy [4, 5].

Solid waste collection scheduling is an integral part of a waste management model. It is generally regarded as a form of vehicle routing problem (VRP) which seeks to minimize the trucks used and the distance traversed. A VRP optimization algorithm requires information about the bin locations, disposal sites, number of available vehicles and their capacity, to get an optimal solution. Thus a prior investigation on the waste accumulation rate of the nodes in a network is useful to set the collection priority for the nodes [6]. The frequency of garbage collection of a node is also influenced by routing time and distance traversed. A long gap between waste collections may leave the waste to rot and contaminate the bins and their surroundings. However, visiting the nodes too frequently is not economical.

In this paper, an image-based collection scheduling of a node with 3 waste bins is considered. Images of the bins are analyzed at a regular interval using an overhead camera. First, the positions of the three bins are detected. Then, the waste level of each bin is determined using Laws Masks and a set of SVM classifiers. The classification result was compared with those of k nearest neighbor (k-nn) and multilayered perceptron (MLP) classifier. The waste levels of the bins are used as observations to an HMM that schedules the optimum collection time for the node. The remainder of the article is outlined as follows. A brief literature review on works related to waste collection, scheduling and routing is presented in the next section. This is followed by the methodology of the proposed method where details of the steps involved are elaborated. Then experiments performed using the proposed method on a custom database are described. Finally, a conclusion is drawn.

## Literature review

With the advancement of technology, compact sensors have been introduced for various applications including solid waste level detection [7]. Optical, capacitive, proximity and weight sensors have been used to gauge the amount of waste in bins [8–12]. Some solid waste systems have been integrated with communication networks like the internet of things [13]. Some attempts have been made to use computer vision and image processing techniques to classify waste level in bins. Gabor filter, gray level aura matrix, and Sobel edge features have been extracted and used with k-nn, multilayer perceptron (MLP) and support vector machine classifiers to classify waste level in bins [14–16].

Laws energy masks were introduced to classify textures but since their introduction, they have been used for various applications [17–19]. Normally, features are extracted by convolving an image with selected laws masks and used with a classifier. These features have been shown to be robust and highly discriminating. In this work, Laws masks are used to detect bin openings and the waste level in the bins. A support vector machine (SVM) is a powerful binary classifier that is used to split objects into two classes [20]. An SVM is fast, accurate and robust to noisy data [21]. Given a mixed dataset containing data of two classes, the classifier creates a hyperplane that segregate data of the two classes [22]. An SVM uses a kernel function like Gaussian, sigmoid or radial function to classify data with a nonlinear distribution. It is found that the accuracy of SVMs with a radial basis function (RBF) kernel is very competitive [23]. Meanwhile a hidden Markov model (HMM) is a statistical tool that can be used for decision making. The advantage of using a HMM is that the same set of observations can produce a different current state depending on the previous state. Details of the theory and implementation of HMM can be found in [24]. In our experiments, both the SVM and HMM were implemented using MATLAB software packages.

Solid waste collection optimization is analogous to vehicle routing problem (VRP) which requires the optimization of routes traversed by some vehicles that serve a group of sites or customers. Nuortio et al. adapted the VRP to solve a municipal solid waste problem involving waste collection scheduling and vehicle routing [23]. Information about bin location coordinates and distance matrices were included in their system to optimize the time as well as the cost of vehicle routing. Shih and Chang, developed a two-phase approach that optimizes the routing and collection scheduling of solid waste. In the first phase, a route was assigned to each vehicle while in the second phase mixed integer programming was employed to assign collection scheduling. A graphical user interface was also developed for the system [25].

Beltrami and Bodin [26], Tung and Pinnoi [27], Angelelli and Speranza [28] and Teixeira et al. [29] analyzed waste collection scheduling of several collection sites of different collection frequencies. Thus, routes and day of collections must be optimally defined. Household waste can be modeled as a capacitated arc routing problem (CARP) in which every street in a city is represented as an arc in a graph. Naturally all arcs must be visited to collect waste [30–32]. However, commercial waste collection is often regarded as a VRP with time windows because customers often impose windows for collection [32, 33]. McLeod et al. investigated the benefits of collecting household and commercial waste together rather than separately [34]. Besides collection routing, much effort has also been channeled to the design of recycling networks [35–38].

Johansson et al, investigated the efficiency of various routing and scheduling approaches in handling real-time bin data of some recycling stations [39]. The study supports the use of bin level sensors to reduce operating costs and collection. Various sensors have been proposed by researchers to detect garbage level in bins and they include infrared LED sensors [39], capacitive moisture sensor [40], point-level capacitive sensor [41] and optical sensor [42]. However, many believe that the use of sensors is impractical due to the cost of installation and maintenance. Furthermore, some sensors will only work properly under certain conditions. For instance, capacitive sensors are affected by humidity and they do not work well during rainy season.

Rovetta et al. [43], Vicentini et al. [44] and Zhu et al. [45] developed garbage collection systems based on garbage level which is automatically determined by the difference of consecutive image frames captured over the bin. The weakness of methods that rely on difference images is the requirement that the position of the bin is always fixed relative to the camera so that the same region of interest (ROI) is always captured. If the bin or the camera is shifted or rotated, a different ROI will be captured that amounts to misinterpretation. This requirement is restrictive for real time implementation [14].

Existing waste collection route optimization approaches use prior information on waste generation at various nodes to determine current collection scheduling. To the best of our knowledge, the waste levels of bins at a node have never been explicitly used to set the garbage collection time of the node. Therefore, the idea of using the waste levels of garbage bins at a node to determine its collection time is explored in this study.

Visiting a node at the right time prevents unnecessary trip to the disposal site and its contamination by rotten garbage, thus saving money and time. This paper presents an HMM based collection time scheduling of a node with three bins using their waste levels. By assuming that waste starts to contaminate 3 days after it is dumped, the maximum collection period of a node is set to 3 days after rubbish is first deposited in any of the bin. It is observed that waste materials do not necessarily have linear edges. So, four laws masks of different characteristics are employed to detect the presence of waste in the bins.

## Methodology

The collection of a three bin node was scheduled in three steps: bin detection, classification and scheduling. First, an image of a disposal site, called a node, is analyzed to locate the three bins. Keep in mind that the bins might have been shifted, rotated or occluded by trash. The strategy is to locate each bin by its opening, an area surrounded by four corners. Thus, the first step is to identify potential bin corners using Hough transform. For each bin corner candidate, an associated bin opening area is established. Then features are extracted from the rim of the area to determine whether it is one of the three bin openings. Once the three bin openings are located, a set of features are extracted from every bin opening by a set of Laws masks. Using these features and a set of SVMs, the waste level of each bin is determined consecutively. Finally, an HMM takes the waste levels of the three bins as an observation and depending on its previous state, changes its current state. The current state of the HMM is the number of days before the next waste collection for the node.

### Database

**Fig 1:**
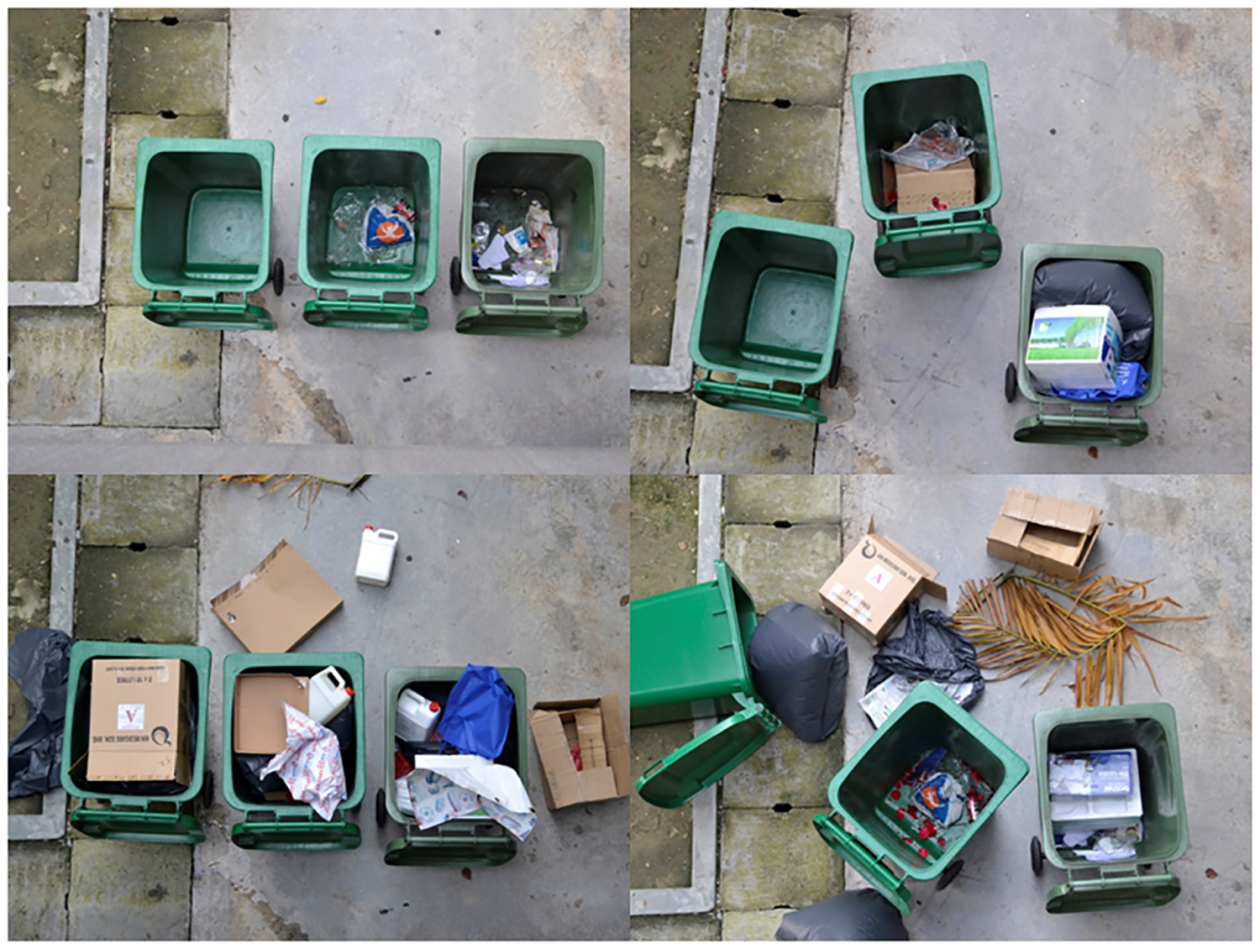
Samples of bin images. Four separate images from database. Each image is showing a solid waste bin node with three bins.

There are 200 images in the custom database and the size of each image is 800 × 600 pixel. The images were taken with a low-cost camera located above the disposal site. All the images contain 3 waste bins that are rectangular with an opening area that is approximately 300 × 300 pixels. 100 images from the database are utilized for training and the remaining 100 images for testing. Fig 1 shows some bin images from the database.

### Corner candidate detection

Given a training or test image, the first thing to do is to extract edge information from it using Canny Edge Operator. Then Hough Transformation is performed on the edge image to find straight lines that are present in the image. Some of these straight lines belong to the edge of the bins we wish to detect while others belong to the trash or background objects. For each detected line, two parameters are calculated. They are the length of the vector from the origin to the nearest point on the line and the angle that the vector forms with the horizontal axis at the origin. The two parameters, called *ρ* and *θ* are used in an equation that represents the line as follows

ax+by=ρ (1)

where *a* = *cosθ* and *b* = *sinθ*. Of all the detected lines, only forty with the highest quantity of edge pixels that fall on them are considered as possible edges of the bin openings. The number of lines to be analyzed is further reduced by excluding lines that are not long enough to be bin edges from further processing. Next, the orthogonality of every pair of lines represented by (*a*_1_, *b*_1_, *ρ*_1_) and (*a*_2_, *b*_2_, *ρ*_2_) is tested by multiplying *a*_1_ by *a*_2_ and *b*_1_ by *b*_2_ and adding the products. If the total is close to zero they are regarded as orthogonal, and the coordinate of their intersection point (*x*, *y*) is calculated by

$$x=\frac{\rho_{1}b_{2}-\rho_{2}b_{1}}{a_{1}b_{2}-a_{2}b_{1}}$$ (2)

$$y=\frac{\rho_{1}a_{2}-\rho_{2}a_{1}}{a_{2}b_{1}-a_{1}b_{2}}$$ (3)

The intersection points of the orthogonal lines represent possible locations of the bin corners. Since the bins are rigid objects, when they are rotated, their corners will also rotate by the same amount. The angle of rotation for each corner candidate is acquired from the parameters of the two orthogonal lines that form it. Finally, areas of the three bin openings are located and classified.

### Bin opening area classification

Besides the bins, rubbish with angled corners also produce corner candidates. Thus, we need to distinguish bin corners from rubbish corners. Each corner candidate is assumed to sit at a corner of a bin opening. Then a square area approximately the size of the bin opening is constructed using the lines that form the corner candidate as they coincide with the edges of the opening. Based on the angles of the two lines, the degree of rotation of the opening area is easily calculated. Once the angle of rotation of the area is identified, it is unrotated so that it is upright. Then the edge of the square area is convolved with *L*5*E*5 Laws mask to extract four features which are used to determine whether the area is a true bin opening or not.

The idea is that the appearance of the edge is not affected by the waste in the bin. Even if one or two edges are occluded by rubbish, the remaining edges are still visible enough to indicate the bin opening. We allocate an area of 25 × 300 pixels along the four edges of the bin opening for convolution with *L*5*E*5 mask. The four extracted features are then used to classify the area as a true bin opening or not. The number of bin openings in the image is limited to three but if less than three openings are found then the missing bins are considered toppled over or removed from the disposal site.

### Feature extraction

Laws [17] developed a set of one-dimensional filters called level, edge, spot and ripple. When convolved with each other, a number of square masks are obtained but the most useful ones are *L*5*E*5, *E*5*S*5, *R*5*R*5 and *L*5*S*5. The mask are rotated to four directions of (0°, 45°, 90° and 135°) and convolved with the entire bin opening area of 300 × 300 pixels. The masks and their rotated forms are shown in Table 1. For each mask, only the result of convolution with the direction that gives the maximum energy is taken as a feature. Therefore for four masks, four features are obtained from the bin opening area.

**Table 1:** The four laws masks and their rotated forms.

	L5E5					E5S5					R5R5					L5S5				
0°	-1	-2	0	2	1	1	0	-2	0	1	1	-4	6	-4	1	-1	0	2	0	-1
	-4	-8	0	8	4	2	0	-4	0	2	-4	16	-24	16	-4	-4	0	8	0	-4
	-6	-12	0	12	6	0	0	0	0	-2	6	-24	36	-24	6	-6	0	12	0	-6
	-4	-8	0	8	4	-2	0	4	0	-2	-4	16	-24	16	-4	-4	0	8	0	-4
	-1	-2	0	2	1	-1	0	2	0	-1	1	-4	6	-4	1	-1	0	2	0	-1
45°	0	2	1	4	6	-2	0	1	2	0	6	-4	1	-4	6	2	0	-1	-4	-6
	-2	0	8	12	4	0	-4	0	0	-2	-4	-24	16	-24	-4	0	8	0	0	-4
	-1	-8	0	8	1	1	0	0	0	-1	1	16	36	16	1	-1	0	12	0	-1
	-4	-12	-8	0	2	2	0	0	4	0	-4	-24	16	-24	-4	-4	0	0	8	0
	-6	-4	-1	-2	0	0	-2	-1	0	2	6	-4	1	-4	6	-6	-4	-1	0	2
90°	1	4	6	4	1	1	2	0	-2	-1	1	-4	6	-4	1	-1	-4	-6	-4	-1
	2	8	12	8	2	0	0	0	0	0	-4	16	-24	16	-4	0	0	0	0	0
	0	0	0	0	0	-2	-4	0	4	2	6	-24	36	-24	6	2	8	12	8	2
	-2	-8	-12	-8	-2	0	0	0	0	0	-4	16	-24	16	-4	0	0	0	0	0
	-1	-4	-6	-4	-1	1	2	0	-2	-1	1	-4	6	-4	1	-1	-4	-6	-4	-1
135°	6	4	1	2	0	0	-2	-1	0	2	6	-4	1	-4	6	-6	-4	-1	0	2
	4	12	8	0	-2	2	0	0	4	0	-4	-24	16	-24	-4	-4	0	0	8	0
	1	8	0	-8	-1	1	0	0	0	-1	1	16	36	16	1	-1	0	12	0	-1
	2	0	-8	-12	-4	0	-4	0	0	-2	-4	-24	16	-24	-4	0	8	0	0	-4
	0	-2	-1	-4	-6	-2	0	1	2	0	6	-4	1	-4	6	2	0	-1	-4	-6

### Bin level classification

The four features extracted using Laws masks from each bin area are fed into two SVM classifiers that determine whether the bin is empty, partially full or full. Since there are possibly three bins in each image, the waste level of each bin is determined sequentially by the two SVMs using the four features from each bin area. But if a bin is toppled over, no feature is extracted from it. Four additional features are extracted from the area outside the bins using the same Laws masks.

**Fig 2:**
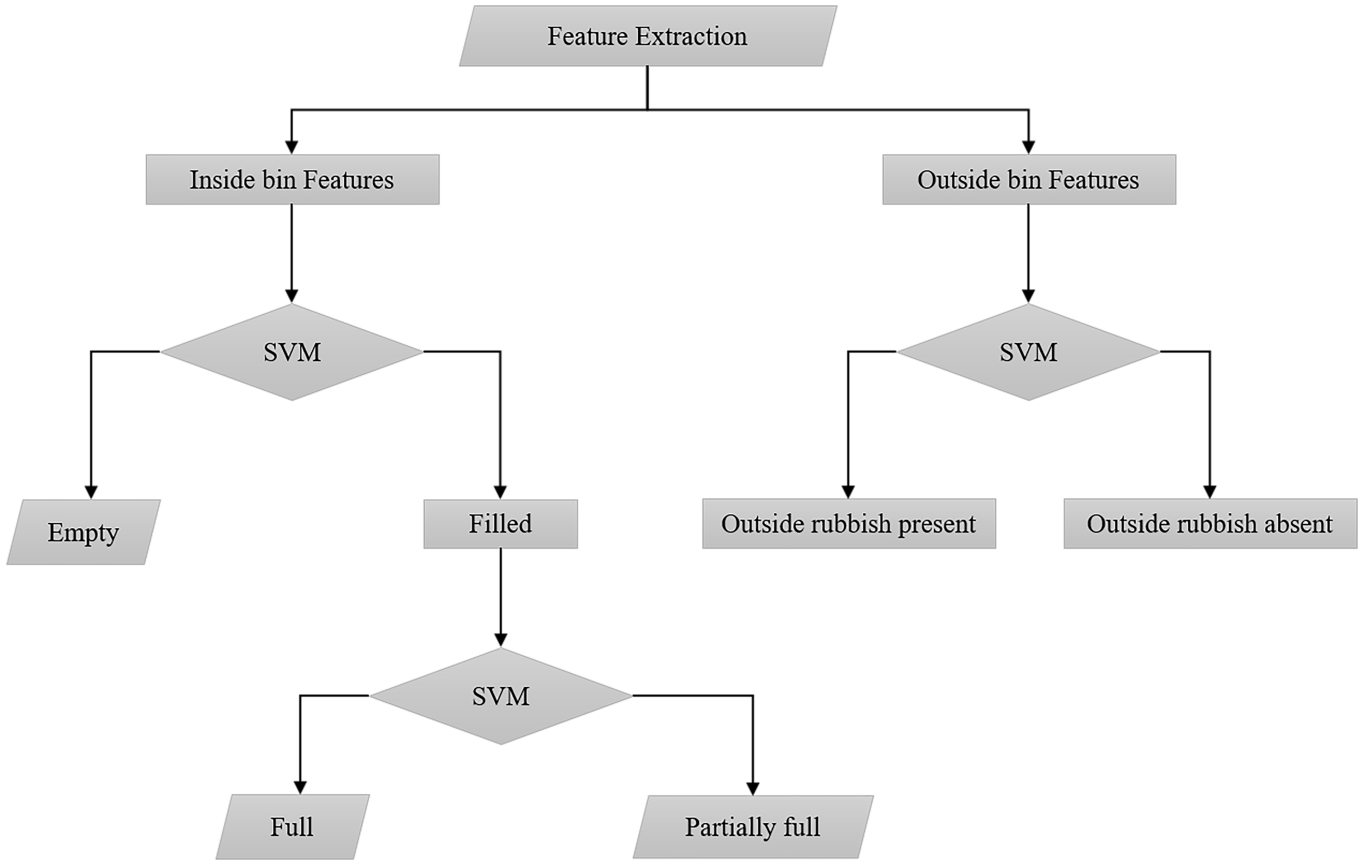
Bin level classification. Three separate SVM classifiers were used to classify the bin levels. One SVM classify the outside bin area to detect the presence of rubbish outside. The levels of wastes were detected by classifying the insides of bins using two SVMs.

In our experiments, Radial Basis function is selected as the kernel for the SVM classifiers used in the waste level classification. As a supervised classifier, an SVM requires training. For each detected bin, four input features are obtained from its opening and used by the first SVM to decide whether there is any garbage in the bin or not. If there is no garbage found in the bin, it is considered *empty*. Otherwise the four features are passed to the second SVM to determine whether the bin level is either partially full or full. This process is repeated for every bin detected. The four features from the area outside of the three bins are assigned to the third SVM to examine whether waste is thrown outside the bins. The classification process is illustrated in Fig 2.

### Scheduling

The collection time of the node depends on the state of the HMM. The state is in the form of the number of days before collection. It can be indeterminate, 3 days, 2 days, 1 day or immediate. If all bins are empty, an indeterminate number of days before collection is assigned to the node. As soon as rubbish is deposited in a bin, the state is assigned to three days as we assume that it takes three days for the rubbish to start rotting and contaminating. This choice is ad hoc and can be lengthened if necessary. Then the number of days until collection will decrease every 24 hours and the countdown is not affected by the amount of rubbish in the three bins unless all three bins become full. Suffice to say that the state of the node is dictated by the time the first rubbish is deposited. If the bin containing the first rubbish becomes full in less than three days, but at least one bin is not full, the countdown continues. If all three bins are full before three days then the state of the node becomes immediate as there is no more room left for new rubbish to fill up. When rubbish is collected and all bins become empty, the node is reset to indeterminate. The rules are summarized as follows.

1. The current state of a node is the number of days remaining before rubbish collection. After 24 hours has elapsed, the number of days is reduced by one unless all bins become full or empty.
2. The previous state was the state of the node 24 hours earlier.
3. The next state is the expected state of the node 24 hours later unless all bins become full or empty.
4. Once rubbish is deposited in one or all bins, the current state changes from indeterminate to 3 days and countdown begins.
5. If all bins are full then current state is indeterminate regardless of previous state.
6. If all bins are full then current state is immediate regardless of previous state.
7. If one or more bins are missing or toppled over then current state is immediate regardless of previous state.

The observation and state transition are shown in Table 2. The state starts from *indeterminate* where all the 3 bin levels are empty. If rubbish is deposited in one or more bins but they are not full, then the state is set to three days and countdown begins. If any bin is missing or toppled over then the state jumps to *Immediate* from any state. This is also true if all bins are full. We do not consider the odd case of rubbish littered outside the bin when there is still an empty or partially full bin. If this unusual case is to be considered, it could be detected by observing the output of the SVM that takes the features from the area outside of the bins.

**Table 2:** Nulla mi mi, venenatis sed ipsum varius, volutpat euismod diam.

Observation	Previous State	Current State	Next State after 24 hours
All bins are empty	Any	Indeterminate	Indeterminate
Rubbish is first deposited in one or more bins but at least one bin is not full. Current state is set to 3 days and it continues until the next 24 hours elapses.	Indeterminate	3 days	2 days
48 hours after rubbish is first deposited. More rubbish may be added but at least one bin is still not full. This state continues until the next 24 hours elapses.	2 days	1 day	Immediate
72 hours after rubbish is first deposited. More rubbish may be added but at least one bin is still not full. This state continues until rubbish is collected from the three bins.	1 day	Immediate	Immediate
All 3 bins are full or at least one bin is missing or toppled over.	Any	Immediate	Immediate

Phasellus venenatis, tortor nec vestibulum mattis, massa tortor interdum felis, nec pellentesque metus tortor nec nisl. Ut ornare mauris tellus, vel dapibus arcu suscipit sed.

## Experiments

Altogether there are 200 images of 800 × 600 pixels that contain three 120L sized waste bin that are empty, partially full or full of rubbish. The opening of each bin is square and its size is roughly 300 × 300 pixels. Half of the images are used for training the SVM classifiers and the rest are for testing the performance of the method. Given a color test image, it is converted to greyscale before undergoing edge detection using canny operator.

**Fig 3:**
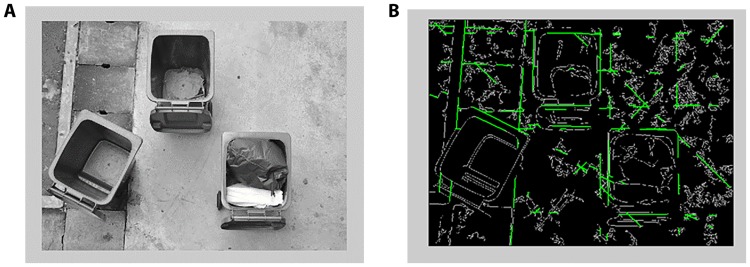
Bin edge lines in binary image. (A) The input image. (B) The detected lines using the Hough transform.

Then Hough transform is applied to the binary image to detect lines in it as illustrated in Fig 3. The objective is to locate the three bin openings, each has four sides and four corners. Each corner is represented by the intersection of two orthogonal lines. The intersecting lines also provide the position and orientation of the corner that they form. Only 30 corner candidates are considered in each image as a maximum of 12 candidates belong to the bins and the rest come from the trash.

**Fig 4:**
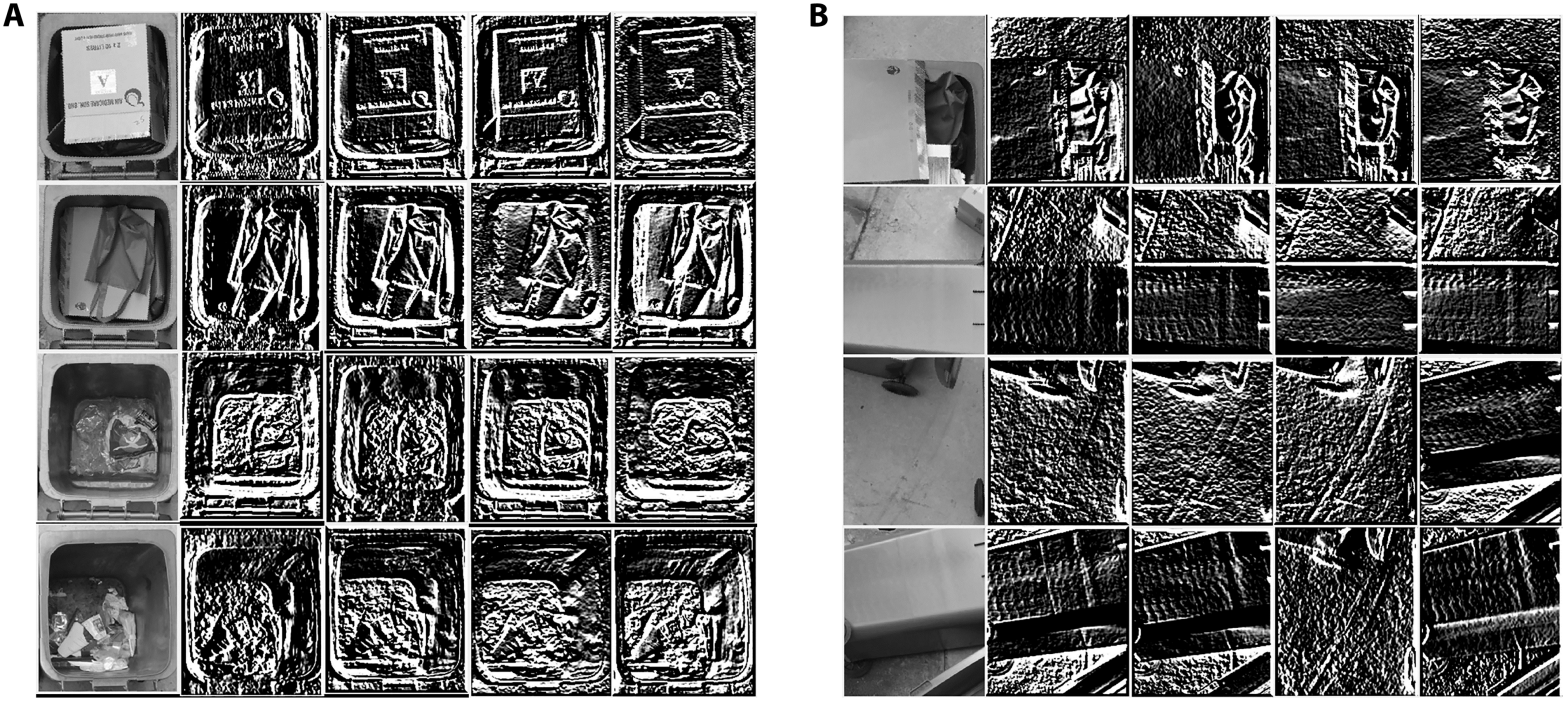
Bin opening candidates and their L5E5 features. (A) The selected candidates. (B) The rejected candidates. The columns of each row from left to right are the candidate, 0°, 45°, 90°, and 135° *L*5*E*5 mask convolved images.

Each corner candidate is assumed to sit at a corner of a bin opening. Then a square area approximately the size of the bin opening is constructed using the lines that form the corner candidate as they lie on the edges of the opening area. Based on the angles of the two lines, the bin area is unrotated so that it is upright. Then the four edges of the bin area, the size of each is 25 × 300, is convolved with the *L*5*E*5 Laws mask to extract four features which are used to determine whether the area is a true bin opening or not. A maximum of three bin areas are expected but if there are less than three bin areas found, the remaining is considered missing or toppled over. An SVM classifier is trained by samples from the 100 training images to classify correct and wrong bin areas. Then the other 100 images are used to test whether the proposed algorithm can detect all bin openings in the test images. It is found that all bins in the test images are detected correctly. Few examples of selected and rejected bin opening areas and their extracted features are shown in Fig 4.

**Fig 5:**
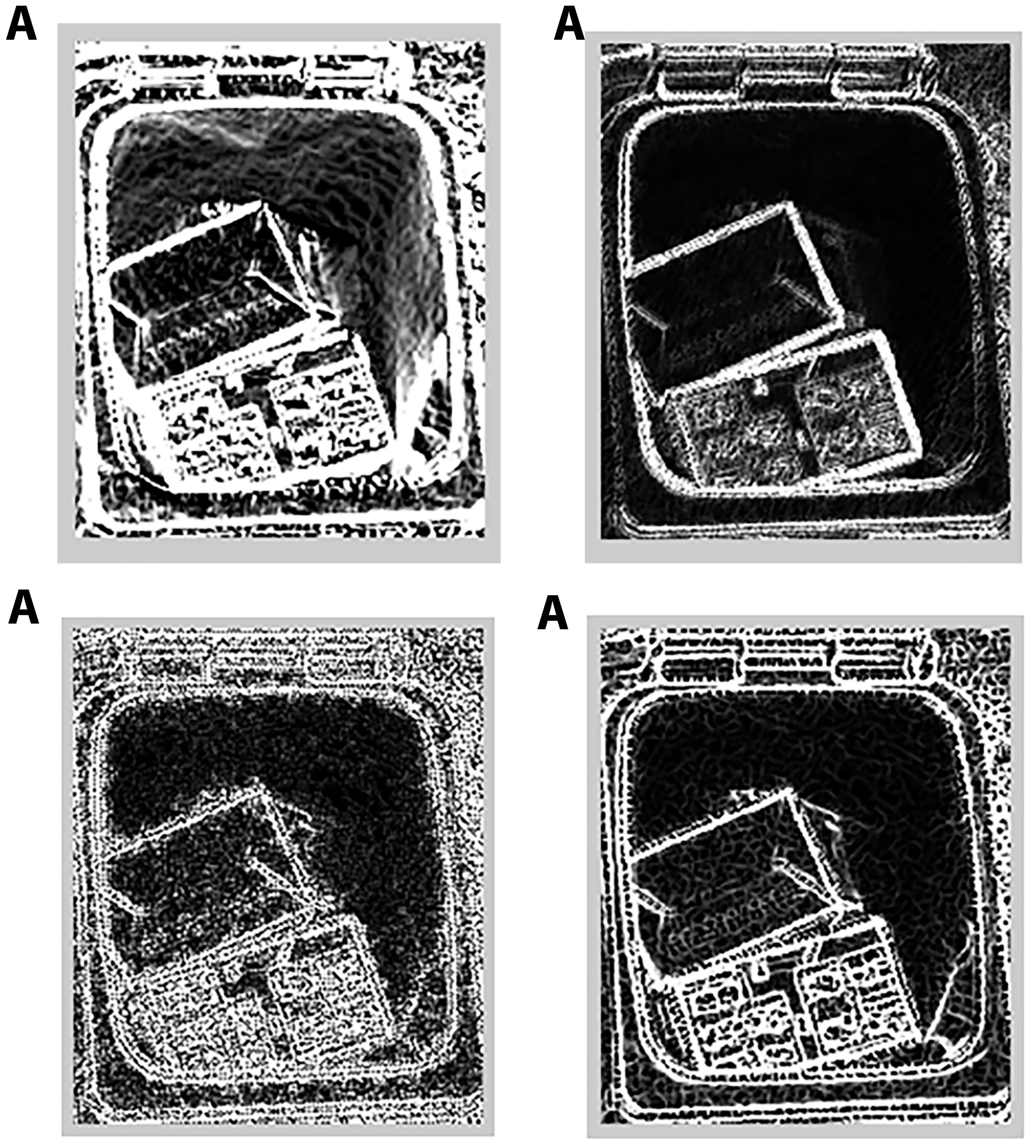
Feature extraction of bin area. The convovlved images of a bin area with laws masks (A) *L*5*E*5, (B) *E*5*S*5, (C) *R*5*R*5, and (D) *L*5*S*5.

Once the bin areas are located, they are convolved with the four laws masks of *L*5*E*5, *E*5*S*5, *R*5*R*5 and *L*5*S*5 at 0°, 45°, 90° and 135° orientations. However, only the orientation that gives the maximum output is kept as a feature for a mask. In total, four features are obtained from each bin area. Examples of the results of the convolution of a bin area with the four masks are shown in Fig 5. For waste level classification, an SVM classifier takes the four features from each bin area and decides whether the bin is filled or empty. If the bin is filled, another SVM will evaluate the four said features to reach a conclusion that the bin is partially full or full. Lastly, another SVM takes four features extracted from the outside of the bins to detect the presence of garbage outside of the bins. The k-nn classifier was trained using same four-dimensional feature set to identify 10 k points for each class to classify new sample. The nearest neighbor is selected by minimum Euclidean distance between the sample and the k points. The same feature set was used to train MLP. The input layer has 4 nodes for four feature vector. The hidden layer was optimized to produce logistics using sigmoid activation function. The output layer had three nodes for three classes.

## Results and discussion

**Fig 6:**
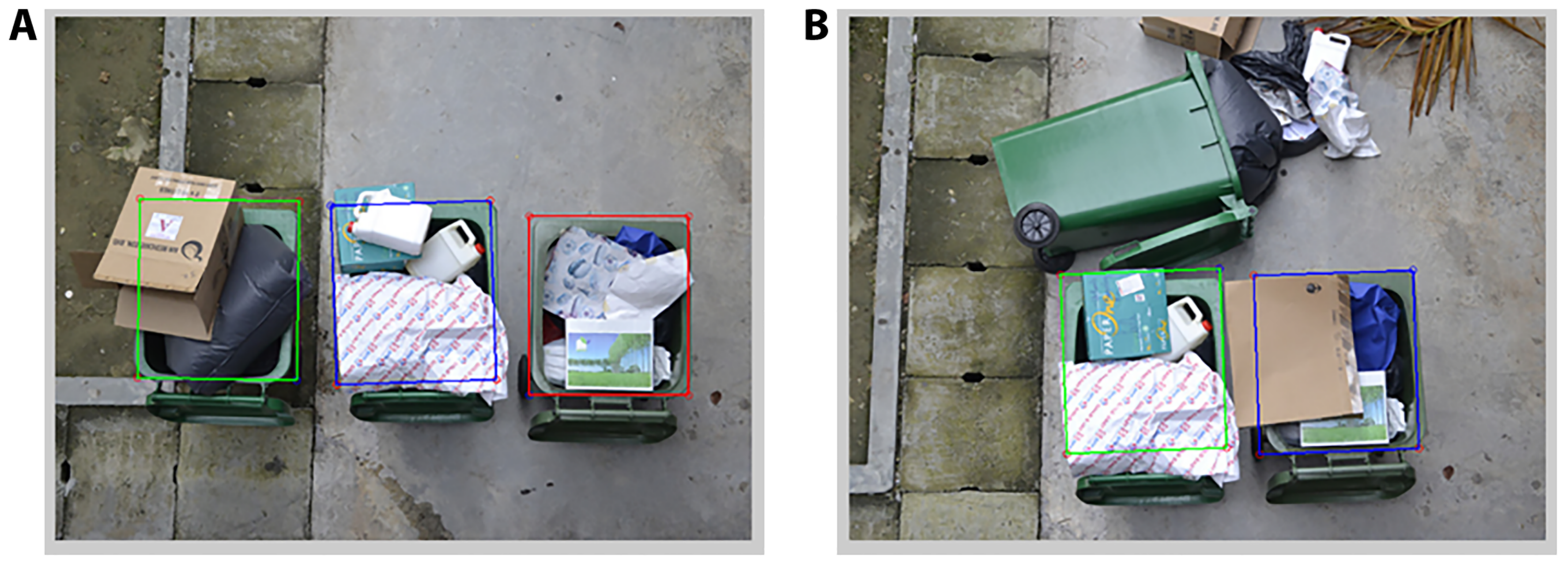
The results of bin localization of three test images. (A) Three bin areas are detected by green, blue and red rectangles. (B) Only two bins, green and blue rectangles are detected because the third bin was toppled over.

The effectiveness of the algorithm in detecting the bins, determining the waste level of each bin and the choosing the collection time of the node based on the rubbish levels of the bins were assessed separately. In locating the bin openings in the test images, the proposed method managed to locate all standing bins correctly. If there were less than three bins detected, the method could recognize that one bin was missing or toppled over. Therefore, the accuracy of the bin detection is 100%. A few examples of selected and rejected bin openings are shown in Fig 6.

The k-nn, MLP and SVM classifiers were trained using 120 empty, 92 partially full and 75 full bin images. These images were cropped manually from the 100 training images. All of the classifiers were trained using the same four features extracted by laws masks. Then the bin openings in the 100 test images were detected and their waste levels were classified using the trained classifiers. In classifying the waste level of each detected bin, the k-nn and MLP classifiers record 93% and 98% classification rate respectively. On the other hand, only one empty bin was misclassified as partially full by SVM classifier and this amounts to a classification rate of 99.73%. Upon close inspection, we found that the empty bin that was misclassified was particularly dirty. The overall results of bin location detection and waste level classification for the 100 test images are given in Table 3.

**Table 3:** Bin level detection accuracy.

Classes	k-nn			MLP			SVM		
	TP	FN	Sensitivity	TP	FN	Sensitivity	TP	FN	Sensitivity
Empty	118	5	95.93%	120	3	97.56%	122	1	99.19%
Partial	84	7	92.31%	88	3	96.70%	91	0	100.00%
Full	72	8	90.00%	80	0	100.00%	80	0	100.00%
Overall accuracy			92.75%			98.09%			99.73%

TP = True Positive, FN = False Negative, and NA = Not Applicable.

For the collection time or garbage collection scheduling of the node, the bins and their waste levels need to be monitored continuously. Their presence, upright position and waste levels were the observations of the HMM. The HMM parameters were calculated by Forward-Backward algorithm and optimized by Baum-Welch algorithm. The states were decoded by Viterbi algorithm. The rate of successful estimated states was considered as the score of the model and the model was optimized until the score reached 100%. The confusion matrix of the state of the HMM based on its observations is shown in Table 4. The average accuracy of the collection time of the node is 100%.

**Table 4:** Confusion matrix of state estimation.

Conditions	Indeterminate	3 days	2 days	1 day	Immediate
Indeterminate	25	0	0	0	0
3 days	0	14	0	0	0
2 days	0	0	10	0	0
1 day	0	0	0	21	0
Immediate	0	0	0	0	30

## Conclusion

This paper introduces a waste collection scheduling of a single node with three bins based on HMM observations associated with the capacity of the bins to contain waste and the condition of the waste. Laws masks were used to extract four features from the bin opening of each bin. Using SVM classifiers and the extracted features, the waste level of the bins were classified into empty, partially full or full. Based on its previous state and the waste levels of the bins (observations), the current state of the HMM is determined. The proposed system was trained using 100 training images. Then it was tested on 100 test images of three waste bins that might be shifted, rotated, occluded or toppled over. The bins also contained garbage of various levels. The method obtained bin detection, waste level classification and collection day scheduling rates of 100%, 99.73%, and 100% respectively. The promising results show that the work can be expanded to deal with many nodes having more than three bins.
